# α-Ba_2_P_2_O_7_

**DOI:** 10.1107/S1600536810043539

**Published:** 2010-10-31

**Authors:** Driss Zakaria, Fatima Erragh, Abdelghani Oudahmane, Malika El-Ghozzi, Daniel Avignant

**Affiliations:** a Laboratoire de Physico-Chimie des Matériaux, Université Chouaib Doukkali, Faculté des Sciences BP 20, 24000 El Jadida, Morocco; b Université Blaise Pascal, Laboratoire des Matériaux Inorganiques, UMR CNRS 6002, 24 Avenue des Landais, 63177 Aubière, France

## Abstract

Single crystals of α-Ba_2_P_2_O_7_, dibarium diphosphate, were obtained by solid-state reaction. The ortho­rhom­bic structure is isotypic with α-Sr_2_P_2_O_7_ and is the second polymorph obtained for this composition. The structure is built from two different BaO_9_ polyhedra (both with *m* symmetry), with Ba—O distances in the ranges 2.7585 (10)–3.0850 (6) and 2.5794 (13)–2.9313 (4) Å. These polyhedra are further linked by sharing corners along [010] and either edges or triangular faces perpendicularly to [010] to form the three-dimensional framework. This polyhedral linkage delimits large channels parallel to [010] where the P_2_O_7_ diphosphate anions are located. These groups (symmetry *m*) are characterized by a P—O—P angle of 131.52 (9)° and an eclipsed conformation. They are connected to the BaO_9_ polyhedra through edges and corners.

## Related literature

Besides crystals of the title compound, crystals of the hexa­gonal polymorph σ-Ba_2_P_2_O_7_ were obtained (ElBelghitti *et al.* 1995 ▶). For isotypic structures, see: Hagman *et al.* (1968 ▶); Grenier & Masse (1977 ▶); Barbier & Echard (1998 ▶). For closely related structures, see: Elmarzouki *et al.* (1995 ▶). For polymorphism in Ba_2_P_2_O_7_, see: McCauley & Hummel (1968 ▶); Mehdi *et al.* (1977 ▶); Bian *et al.* (2004 ▶); Kokhanovskii (2004 ▶). For a review of the crystal chemistry of diphosphates, see: Durif (1995 ▶). For applications of alkaline earth diphosphates, see: Pang *et al.* (2009 ▶); Peng *et al.* (2010 ▶). For an independent refinement of the α-Ba_2_P_2_O_7_ structure based on data from a hydro­thermally grown crystal, see: Heyward *et al.* (2010 ▶).

## Experimental

### 

#### Crystal data

Ba_2_P_2_O_7_
*M* *_r_* = 448.62
Orthorhombic, 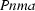

*a* = 9.2875 (1) Å
*b* = 5.6139 (1) Å
*c* = 13.8064 (1) Å
*V* = 719.85 (2) Å^3^
*Z* = 4
Mo *K*α radiation
μ = 11.31 mm^−1^
*T* = 296 K
0.26 × 0.14 × 0.14 mm

#### Data collection

Bruker APEXII CCD diffractometer
Absorption correction: multi-scan (*SADABS*; Bruker, 2008 ▶) *T*_min_ = 0.155, *T*_max_ = 0.205
18034 measured reflections
4304 independent reflections
3726 reflections with *I* > 2σ(*I*)
*R*_int_ = 0.028

#### Refinement

*R*[*F* ^2^ > 2σ(*F* ^2^)] = 0.021
*wR*(*F* ^2^) = 0.046
*S* = 1.07
4304 reflections
62 parameters
Δρ_max_ = 1.59 e Å^−3^
Δρ_min_ = −1.99 e Å^−3^

### 

Data collection: *APEX2* (Bruker, 2008 ▶); cell refinement: *SAINT* (Bruker, 2008 ▶); data reduction: *SAINT*; program(s) used to solve structure: *SHELXS97* (Sheldrick, 2008 ▶); program(s) used to refine structure: *SHELXL97* (Sheldrick, 2008 ▶); molecular graphics: *DIAMOND* (Brandenburg, 1999 ▶) and *ORTEP-3 for Windows* (Farrugia, 1997 ▶); software used to prepare material for publication: *SHELXTL* (Sheldrick, 2008 ▶).

## Supplementary Material

Crystal structure: contains datablocks I, global. DOI: 10.1107/S1600536810043539/wm2413sup1.cif

Structure factors: contains datablocks I. DOI: 10.1107/S1600536810043539/wm2413Isup2.hkl

Additional supplementary materials:  crystallographic information; 3D view; checkCIF report

## Figures and Tables

**Table 1:** Selected bond lengths (Å)

Ba1—O3^i^	2.7585 (10)
Ba1—O2^ii^	2.7591 (10)
Ba1—O4^iii^	2.7978 (13)
Ba1—O2	2.8392 (10)
Ba1—O5	3.0850 (6)
Ba2—O5^iv^	2.5794 (13)
Ba2—O3^ii^	2.7377 (10)
Ba2—O2^iii^	2.8133 (10)
Ba2—O3^v^	2.9094 (10)
Ba2—O4^vi^	2.9313 (4)
P1—O5	1.5067 (13)
P1—O2^vii^	1.5208 (11)
P1—O2	1.5208 (11)
P1—O1	1.6148 (13)
P2—O4^viii^	1.5175 (13)
P2—O3^vii^	1.5236 (11)
P2—O3	1.5236 (11)
P2—O1	1.6012 (14)

Symmetry codes: (i) 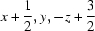; (ii) 
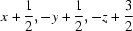
; (iii) 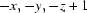; (iv) 
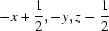
; (v) 
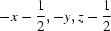
; (vi) 
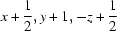
; (vii) 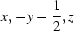; (viii) 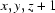.

**Fig. 1.**
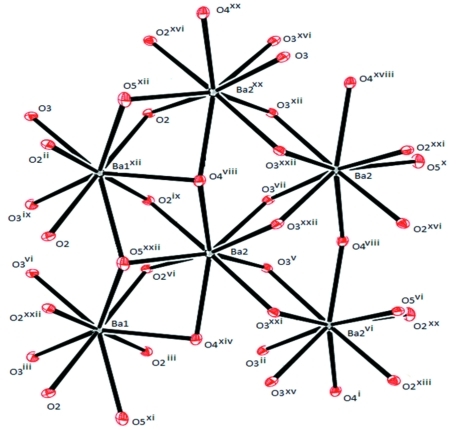
View of the BaO9 polyhedra linkage in α-Ba2P2O7. Displacement ellipsoids are drawn at the 50% probability level. Symmetry codes: (i) x + 1/2, y, -z + 3/2; (ii) x + 1/2, -y + 1/2, -z + 3/2; (iii) -x, -y, -z + 1; (iv) x, -y + 1/2, z; (v) x, y + 1, z; (vii) -x, y + 1/2, -z + 1; (viii) -x - 1/2, -y, z - 1/2; (ix) -x - 1/2, y + 1/2, z - 1/2; (x) x + 1/2, y + 1, -z + 1/2; (xi) x + 1/2, y, -z + 1/2; (xii) x + 1/2, y + 1, -z + 3/2; (xiii) x, -y - 1/2, z; (xiv) x, y, z + 1; (xv) -x - 1/2, -y, z + 1/2; (xvi) x - 1/2, y, -z + 3/2; (xviii) x, y, z - 1; -z + 1/2; (xx) x - 1/2, y, -z + 1/2; (xxi) -x + 1/2, -y, z + 1/2; (xxii) x, y - 1, z;

**Fig. 2.**
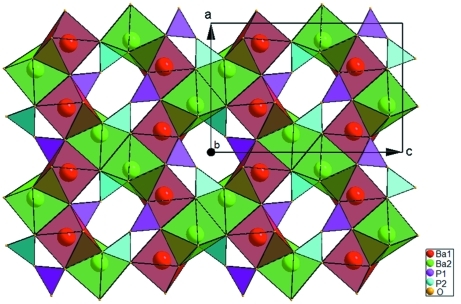
Projection of the α-Ba2P2O7 structure along [010] showing the connections between the P2O7 diphosphate groups and the three-dimensional framework of the BaO9 polyhedra.

## supplementary crystallographic information

### Comment

Due to their potential applications as optical materials (Pang *et al.*, 2009, Peng *et al.*, 2010), alkaline earth diphosphates exhibit at the present time a growing interest. Since the optical properties are strongly related to the crystal structure, the study of polymorphism in these materials is worth investigating. Besides the hexagonal form σ-Ba_2_P_2_O_7_ described by ElBelghitti *et al.* (1995), the title compound is the second polymorph for this composition obtained in the form of single-crystals. The orthorhombic title compound α-Ba_2_P_2_O_7_ is isotypic with α-Sr_2_P_2_O_7_ (Hagman *et al.* 1968; Grenier & Masse, 1977; Barbier & Echard, 1998) and closely related to BaPbP_2_O_7_ (Elmarzouki *et al.*, 1995). The existence of this polymorph has previously been mentioned by Durif (1995) and several other authors (McCauley & Hummel, 1968; Mehdi *et al.*, 1977; Bian *et al.*, 2004; Kokhanovskii, 2004).

The structure of the α-polymorph is built up from two different BaO_9_ polyhedra with Ba—O distances ranging from 2.7585 (10) Å to 3.0850 (6) Å and from 2.5794 (13) Å to 2.9313 (4) Å, respectively. Figure 1 displays details of these coordination polyhedra as well as their linkage by corners, edges or triangular faces to form the three-dimensional framework. This polyhedral linkage delimits large channels parallel to [010] where the P_2_O_7_ diphosphate groups are located (Fig. 2). These groups (symmetry *m*) are characterized by a P—O—P angle of 131.52 (9)° and an eclipsed conformation. They are connected to the BaO_9_ polyhedra through edges and corners. Each oxygen atom of the P_2_O_7_ groups, apart from the O1 bridging oxygen, is bonded to three Ba atoms and one phosphorus atom. During the anisotropic refinement of the displacement parameters of the isotypic α-Sr_2_P_2_O_7_ structure (Barbier & Echard, 1998), the authors observed a very strong anisotropy along the *b* direction for both O5 and O1 atoms located in the (010) mirror plane and interpreted this phenomenon as a possible atomic-scale disorder associated with the local loss of mirror symmetry resulting in a non-centrosymmetric structural arrangement. Such an outstanding feature is still present in our anisotropic structure refinement, although the amplitudes of the atomic displacement parameters are significantly lower, especially for the *U*_22_ component of the O5 atom. If the strong anisotropy of the O1 atom is perfectly understandable because of its bridging role, that of the O5 atom strongly bonded to two Ba1 atoms at 3.0850 (6) Å, one Ba2 atom at 2.5794 (13) Å and one P1 atom at 1.5067 (13) Å is more surprising. However, due to the relative homogeneity of the values of the isotropic displacement parameters of all oxygen atoms, it does not seem necessary to envisage any atomic-scale disorder for the O5 atom in the present case.

Simultaneous with our refinement, an independent study of the α-Ba_2_P_2_O_7_ structure from a hydrothermally grown crystal was reported by Heyward *et al.* (2010). The results of both refinements in terms of geometric parameters are the same within the threefold standard deviation.

### Experimental

Single crystals of the title compound have been obtained during the study of the phase relationships in the ternary system Na_2_O—BaO—P_2_O_5_. They were synthesized in the solid state by reacting Na_2_CO_3_, BaCO_3_ and (NH_4_)_2_HPO_4_ in a platinum crucible. A mixture of these reagents taken in the molar ratio 1: 1: 2 was carefully ground in an agate mortar and successively heated at 373 K, 573 K and 773 K for 24 h at each temperature. After a new grinding, the reacting mixture was submitted to a final heat treatment at 973 K for 2 days followed by a slow cooling to room temperature at the rate of 5 K h^-1^. After an abundant washing of the batch with hot water, single crystals of the title compound could have been extracted.

### Refinement

The highest residual peak in the final difference Fourier map was located 0.56 Å from atom Ba1 and the deepest hole was located 0.31 Å from atom Ba2.

### Crystal data

**Table:**

Ba_2_P_2_O_7_	*F*(000) = 792
*M**_r_* = 448.62	*D*_x_ = 4.139 Mg m^−^^3^
Orthorhombic, *P**n**m**a*	Mo *K*α radiation, λ = 0.71073 Å
Hall symbol: -P 2ac 2n	Cell parameters from 7790 reflections
*a* = 9.2875 (1) Å	θ = 3.7–52.2°
*b* = 5.6139 (1) Å	µ = 11.31 mm^−^^1^
*c* = 13.8064 (1) Å	*T* = 296 K
*V* = 719.85 (2) Å^3^	Hexagonal prism, colourless
*Z* = 4	0.26 × 0.14 × 0.14 mm

### Data collection

**Table:**

Bruker APEXII CCD diffractometer	4304 independent reflections
Radiation source: fine-focus sealed tube	3726 reflections with *I* > 2σ(*I*)
graphite	*R*_int_ = 0.028
Detector resolution: 8.3333 pixels mm^-1^	θ_max_ = 52.4°, θ_min_ = 4.5°
ω and φ scans	*h* = −17→20
Absorption correction: multi-scan (*SADABS*; Bruker, 2008)	*k* = −12→10
*T*_min_ = 0.155, *T*_max_ = 0.205	*l* = −30→28
18034 measured reflections	

### Refinement

**Table:**

Refinement on *F*^2^	Primary atom site location: structure-invariant direct methods
Least-squares matrix: full	Secondary atom site location: difference Fourier map
*R*[*F*^2^ > 2σ(*F*^2^)] = 0.021	*w* = 1/[σ^2^(*F*_o_^2^) + (0.0176*P*)^2^ + 0.4317*P*] where *P* = (*F*_o_^2^ + 2*F*_c_^2^)/3
*wR*(*F*^2^) = 0.046	(Δ/σ)_max_ = 0.004
*S* = 1.07	Δρ_max_ = 1.59 e Å^−^^3^
4304 reflections	Δρ_min_ = −1.99 e Å^−^^3^
62 parameters	Extinction correction: *SHELXL97* (Sheldrick, 2008), Fc^*^=kFc[1+0.001xFc^2^λ^3^/sin(2θ)]^-1/4^
0 restraints	Extinction coefficient: 0.0075 (2)

### Special details

**Table:**

Geometry. All e.s.d.'s (except the e.s.d. in the dihedral angle between two l.s. planes) are estimated using the full covariance matrix. The cell e.s.d.'s are taken into account individually in the estimation of e.s.d.'s in distances, angles and torsion angles; correlations between e.s.d.'s in cell parameters are only used when they are defined by crystal symmetry. An approximate (isotropic) treatment of cell e.s.d.'s is used for estimating e.s.d.'s involving l.s. planes.
Refinement. Refinement of *F*^2^ against ALL reflections. The weighted *R*-factor *wR* and goodness of fit *S* are based on *F*^2^, conventional *R*-factors *R* are based on *F*, with *F* set to zero for negative *F*^2^. The threshold expression of *F*^2^ > σ(*F*^2^) is used only for calculating *R*-factors(gt) *etc*. and is not relevant to the choice of reflections for refinement. *R*-factors based on *F*^2^ are statistically about twice as large as those based on *F*, and *R*- factors based on ALL data will be even larger.

### Fractional atomic coordinates and isotropic or equivalent isotropic displacement parameters (Å^2^)

**Table:**

	*x*	*y*	*z*	*U*_iso_*/*U*_eq_	
Ba1	0.159652 (9)	0.2500	0.744870 (6)	0.00801 (2)	
Ba2	0.138138 (9)	0.2500	0.417071 (6)	0.00841 (2)	
P1	−0.04603 (4)	−0.2500	0.81569 (3)	0.00724 (6)	
P2	−0.28072 (4)	−0.2500	0.95778 (3)	0.00722 (6)	
O1	−0.11642 (14)	−0.2500	0.92264 (9)	0.0129 (2)	
O2	−0.09496 (10)	−0.0264 (2)	0.76295 (7)	0.01169 (13)	
O3	−0.35537 (10)	−0.0271 (2)	0.91982 (6)	0.01215 (14)	
O4	−0.27382 (15)	−0.2500	0.06760 (9)	0.0130 (2)	
O5	0.11429 (14)	−0.2500	0.83242 (11)	0.0160 (3)	

### Atomic displacement parameters (Å^2^)

**Table:**

	*U*^11^	*U*^22^	*U*^33^	*U*^12^	*U*^13^	*U*^23^
Ba1	0.00795 (3)	0.00736 (4)	0.00873 (3)	0.000	−0.00047 (2)	0.000
Ba2	0.00856 (3)	0.00891 (4)	0.00776 (3)	0.000	0.00084 (2)	0.000
P1	0.00544 (11)	0.00757 (16)	0.00873 (13)	0.000	0.00010 (9)	0.000
P2	0.00839 (12)	0.00706 (16)	0.00622 (12)	0.000	0.00066 (9)	0.000
O1	0.0088 (4)	0.0198 (7)	0.0100 (4)	0.000	0.0012 (3)	0.000
O2	0.0126 (3)	0.0091 (4)	0.0133 (3)	0.0008 (3)	0.0005 (2)	0.0022 (3)
O3	0.0145 (3)	0.0101 (4)	0.0119 (3)	0.0029 (3)	0.0000 (2)	0.0022 (3)
O4	0.0161 (5)	0.0160 (6)	0.0069 (4)	0.000	0.0003 (3)	0.000
O5	0.0061 (3)	0.0243 (8)	0.0176 (5)	0.000	−0.0008 (3)	0.000

### Geometric parameters (Å, °)

**Table:**

Ba1—O3^i^	2.7585 (10)	P1—O2^xiii^	1.5208 (11)
Ba1—O3^ii^	2.7585 (10)	P1—O2	1.5208 (11)
Ba1—O2^ii^	2.7591 (10)	P1—O1	1.6148 (13)
Ba1—O2^i^	2.7591 (10)	P1—Ba2^iii^	3.3255 (4)
Ba1—O4^iii^	2.7978 (13)	P2—O4^xiv^	1.5175 (13)
Ba1—O2	2.8392 (10)	P2—O3^xiii^	1.5236 (11)
Ba1—O2^iv^	2.8392 (10)	P2—O3	1.5236 (11)
Ba1—O5^v^	3.0850 (6)	P2—O1	1.6012 (14)
Ba1—O5	3.0850 (6)	P2—Ba2^xv^	3.3668 (4)
Ba2—O5^vi^	2.5794 (13)	P2—Ba2^xvi^	3.3812 (2)
Ba2—O3^ii^	2.7377 (10)	P2—Ba2^xvii^	3.3812 (2)
Ba2—O3^i^	2.7377 (10)	O2—Ba1^xvi^	2.7591 (10)
Ba2—O2^iii^	2.8133 (10)	O2—Ba2^iii^	2.8133 (10)
Ba2—O2^vii^	2.8133 (10)	O3—Ba2^xvi^	2.7377 (10)
Ba2—O3^viii^	2.9094 (10)	O3—Ba1^xvi^	2.7585 (10)
Ba2—O3^ix^	2.9094 (10)	O3—Ba2^xv^	2.9094 (10)
Ba2—O4^x^	2.9313 (4)	O4—P2^xviii^	1.5175 (13)
Ba2—O4^xi^	2.9313 (4)	O4—Ba1^iii^	2.7978 (13)
Ba2—P1^iii^	3.3255 (4)	O4—Ba2^xix^	2.9313 (4)
Ba2—P2^viii^	3.3668 (4)	O4—Ba2^xx^	2.9313 (4)
Ba2—P2^xii^	3.3812 (2)	O5—Ba2^xxi^	2.5794 (13)
P1—O5	1.5067 (13)	O5—Ba1^xxii^	3.0850 (6)
			
O3^i^—Ba1—O3^ii^	68.66 (5)	O3^ii^—Ba2—O3^ix^	76.39 (3)
O3^i^—Ba1—O2^ii^	109.06 (3)	O3^i^—Ba2—O3^ix^	104.688 (19)
O3^ii^—Ba1—O2^ii^	72.09 (3)	O2^iii^—Ba2—O3^ix^	94.29 (3)
O3^i^—Ba1—O2^i^	72.09 (3)	O2^vii^—Ba2—O3^ix^	71.99 (3)
O3^ii^—Ba1—O2^i^	109.06 (3)	O3^viii^—Ba2—O3^ix^	50.95 (4)
O2^ii^—Ba1—O2^i^	68.43 (4)	O5^vi^—Ba2—O4^x^	77.53 (3)
O3^i^—Ba1—O4^iii^	141.44 (2)	O3^ii^—Ba2—O4^x^	52.44 (3)
O3^ii^—Ba1—O4^iii^	141.44 (3)	O3^i^—Ba2—O4^x^	118.59 (3)
O2^ii^—Ba1—O4^iii^	73.93 (3)	O2^iii^—Ba2—O4^x^	122.07 (3)
O2^i^—Ba1—O4^iii^	73.93 (3)	O2^vii^—Ba2—O4^x^	71.11 (3)
O3^i^—Ba1—O2	73.87 (3)	O3^viii^—Ba2—O4^x^	131.51 (3)
O3^ii^—Ba1—O2	109.79 (3)	O3^ix^—Ba2—O4^x^	80.73 (3)
O2^ii^—Ba1—O2	177.035 (6)	O5^vi^—Ba2—O4^xi^	77.53 (3)
O2^i^—Ba1—O2	112.59 (4)	O3^ii^—Ba2—O4^xi^	118.59 (3)
O4^iii^—Ba1—O2	103.55 (3)	O3^i^—Ba2—O4^xi^	52.44 (3)
O3^i^—Ba1—O2^iv^	109.79 (3)	O2^iii^—Ba2—O4^xi^	71.11 (3)
O3^ii^—Ba1—O2^iv^	73.87 (3)	O2^vii^—Ba2—O4^xi^	122.07 (3)
O2^ii^—Ba1—O2^iv^	112.59 (4)	O3^viii^—Ba2—O4^xi^	80.73 (3)
O2^i^—Ba1—O2^iv^	177.035 (6)	O3^ix^—Ba2—O4^xi^	131.51 (3)
O4^iii^—Ba1—O2^iv^	103.55 (3)	O4^x^—Ba2—O4^xi^	146.51 (5)
O2—Ba1—O2^iv^	66.25 (4)	O5—P1—O2^xiii^	111.63 (5)
O3^i^—Ba1—O5^v^	146.01 (3)	O5—P1—O2	111.63 (5)
O3^ii^—Ba1—O5^v^	78.63 (3)	O2^xiii^—P1—O2	111.29 (8)
O2^ii^—Ba1—O5^v^	67.45 (3)	O5—P1—O1	105.06 (8)
O2^i^—Ba1—O5^v^	129.77 (3)	O2^xiii^—P1—O1	108.47 (5)
O4^iii^—Ba1—O5^v^	71.89 (3)	O2—P1—O1	108.47 (5)
O2—Ba1—O5^v^	110.43 (3)	P2—O1—P1	131.52 (9)
O2^iv^—Ba1—O5^v^	49.81 (3)	P1—O2—Ba1^xvi^	136.86 (5)
O3^i^—Ba1—O5	78.63 (3)	P1—O2—Ba2^iii^	95.57 (5)
O3^ii^—Ba1—O5	146.01 (3)	Ba1^xvi^—O2—Ba2^iii^	95.66 (3)
O2^ii^—Ba1—O5	129.77 (3)	P1—O2—Ba1	104.14 (5)
O2^i^—Ba1—O5	67.45 (3)	Ba1^xvi^—O2—Ba1	112.16 (4)
O4^iii^—Ba1—O5	71.89 (3)	Ba2^iii^—O2—Ba1	106.55 (3)
O2—Ba1—O5	49.81 (3)	P2—O3—Ba2^xvi^	101.17 (4)
O2^iv^—Ba1—O5	110.43 (3)	P2—O3—Ba1^xvi^	136.36 (5)
O5^v^—Ba1—O5	130.97 (5)	Ba2^xvi^—O3—Ba1^xvi^	111.02 (4)
O5^vi^—Ba2—O3^ii^	110.68 (3)	P2—O3—Ba2^xv^	93.55 (5)
O5^vi^—Ba2—O3^i^	110.68 (3)	Ba2^xvi^—O3—Ba2^xv^	103.61 (3)
O3^ii^—Ba2—O3^i^	69.25 (4)	Ba1^xvi^—O3—Ba2^xv^	106.11 (3)
O5^vi^—Ba2—O2^iii^	74.14 (3)	P2^xviii^—O4—Ba1^iii^	160.15 (8)
O3^ii^—Ba2—O2^iii^	169.56 (3)	P2^xviii^—O4—Ba2^xix^	93.46 (3)
O3^i^—Ba2—O2^iii^	118.44 (3)	Ba1^iii^—O4—Ba2^xix^	92.23 (3)
O5^vi^—Ba2—O2^vii^	74.14 (3)	P2^xviii^—O4—Ba2^xx^	93.46 (3)
O3^ii^—Ba2—O2^vii^	118.44 (3)	Ba1^iii^—O4—Ba2^xx^	92.23 (3)
O3^i^—Ba2—O2^vii^	169.56 (3)	Ba2^xix^—O4—Ba2^xx^	146.51 (5)
O2^iii^—Ba2—O2^vii^	53.01 (4)	P1—O5—Ba2^xxi^	161.87 (9)
O5^vi^—Ba2—O3^viii^	144.15 (3)	P1—O5—Ba1^xxii^	94.30 (3)
O3^ii^—Ba2—O3^viii^	104.688 (19)	Ba2^xxi^—O5—Ba1^xxii^	93.20 (3)
O3^i^—Ba2—O3^viii^	76.39 (3)	P1—O5—Ba1	94.30 (3)
O2^iii^—Ba2—O3^viii^	71.99 (3)	Ba2^xxi^—O5—Ba1	93.20 (3)
O2^vii^—Ba2—O3^viii^	94.29 (3)	Ba1^xxii^—O5—Ba1	130.97 (5)
O5^vi^—Ba2—O3^ix^	144.15 (3)		

Symmetry codes: (i) *x*+1/2, *y*, −*z*+3/2; (ii) *x*+1/2, −*y*+1/2, −*z*+3/2; (iii) −*x*, −*y*, −*z*+1; (iv) *x*, −*y*+1/2, *z*; (v) *x*, *y*+1, *z*; (vi) −*x*+1/2, −*y*, *z*−1/2; (vii) −*x*, *y*+1/2, −*z*+1; (viii) −*x*−1/2, −*y*, *z*−1/2; (ix) −*x*−1/2, *y*+1/2, *z*−1/2; (x) *x*+1/2, *y*+1, −*z*+1/2; (xi) *x*+1/2, *y*, −*z*+1/2; (xii) *x*+1/2, *y*+1, −*z*+3/2; (xiii) *x*, −*y*−1/2, *z*; (xiv) *x*, *y*, *z*+1; (xv) −*x*−1/2, −*y*, *z*+1/2; (xvi) *x*−1/2, *y*, −*z*+3/2; (xvii) *x*−1/2, *y*−1, −*z*+3/2; (xviii) *x*, *y*, *z*−1; (xix) *x*−1/2, *y*−1, −*z*+1/2; (xx) *x*−1/2, *y*, −*z*+1/2; (xxi) −*x*+1/2, −*y*, *z*+1/2; (xxii) *x*, *y*−1, *z*.
